# Supplementary material for: Zinc(II)-Sterol Hydrazone Complex as a Potent Anti-Leishmania Agent: Synthesis, Characterization, and Insight into Its Mechanism of Antiparasitic Action
Source: Pharmaceutics. 2023 Mar 31;15(4):1113. doi: 10.3390/pharmaceutics15041113 (PMC10142724; doi:10.3390/pharmaceutics15041113)
Supplement: Supplementary file 1 [file pharmaceutics-15-01113-s001.zip › pharmaceutics-2301846-supplementary.pdf]

Zinc(II)-Sterol Hydrazone Complex as a Potent Anti-*Leishmania* Agent: Synthesis, Characterization, and Insight into Its Mechanism of Antiparasitic Action

Gonzalo Visbal [1,*], Rodrigo M. S. Justo [2,†], Gabrielle dos Santos da Silva e Miranda [3,†], Sara Teixeira de Macedo Silva [4], Wanderley de Souza [5,6], Juliany Cola Fernandes Rodrigues [3] and Maribel Navarro [2,*]

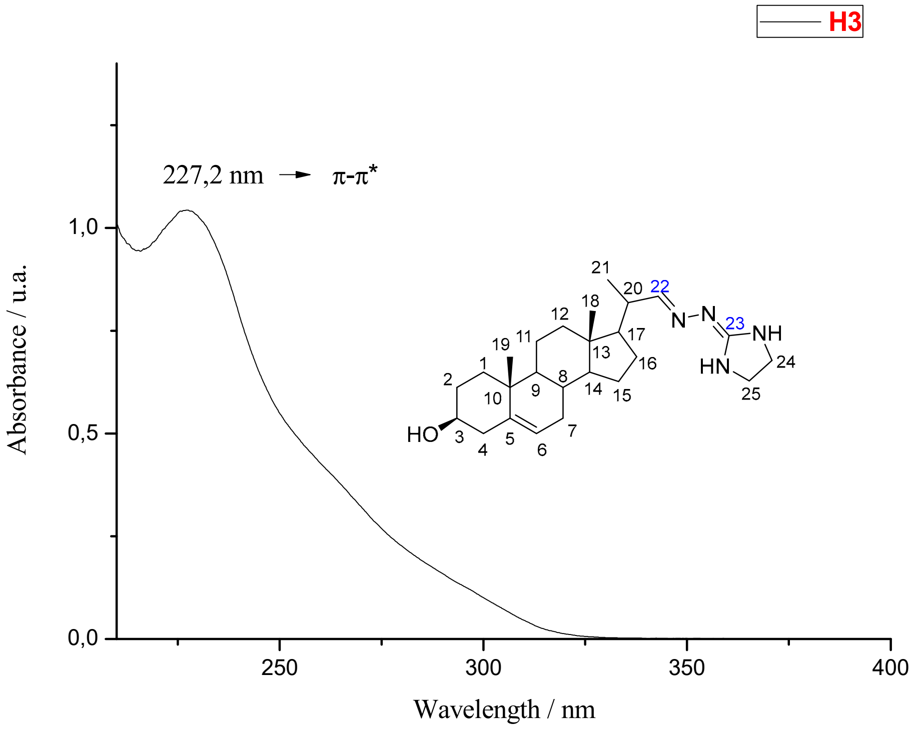

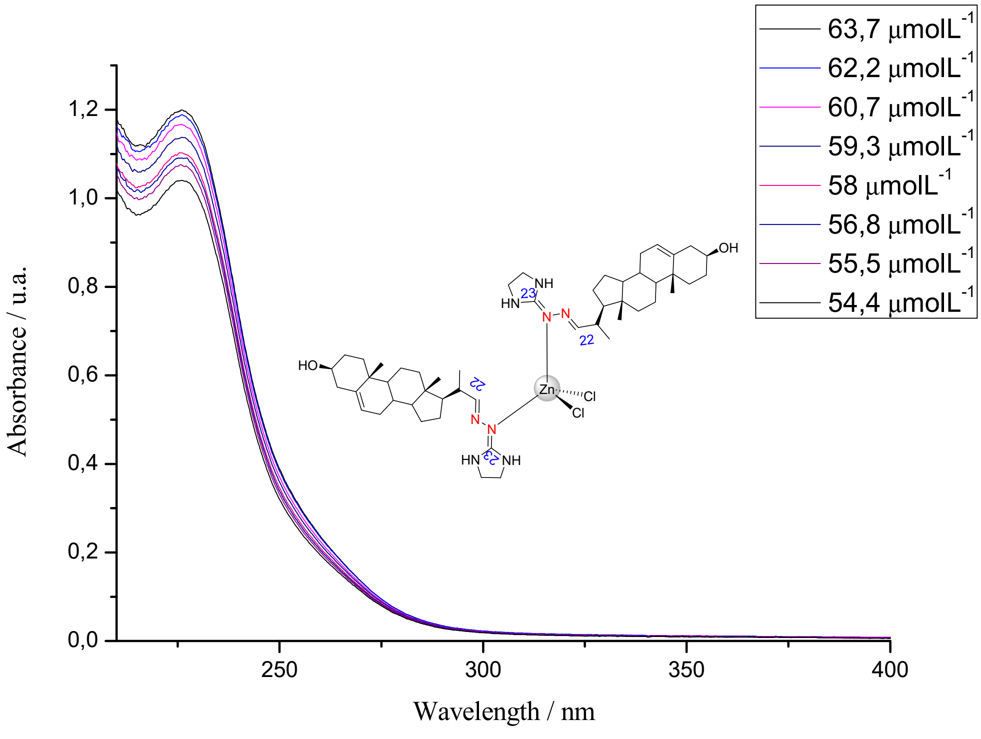

Figure S1. UV absorption spectra of the free ligand **H3** and molar absorptivity spectra for the complex $ZnCl_2$(**H3**)$_2$

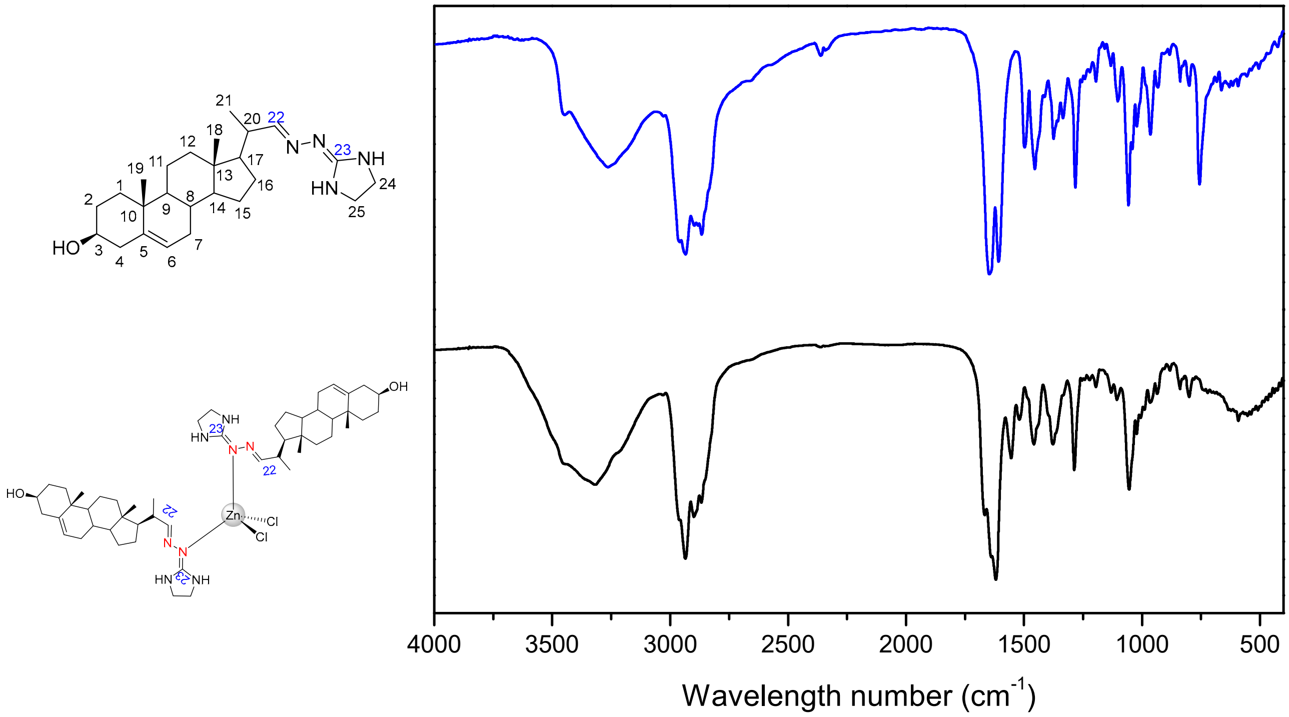

Figure S2. Comparison of the infrared spectra of the free ligand **H3** and the complex $ZnCl_2$(**H3**)$_2$

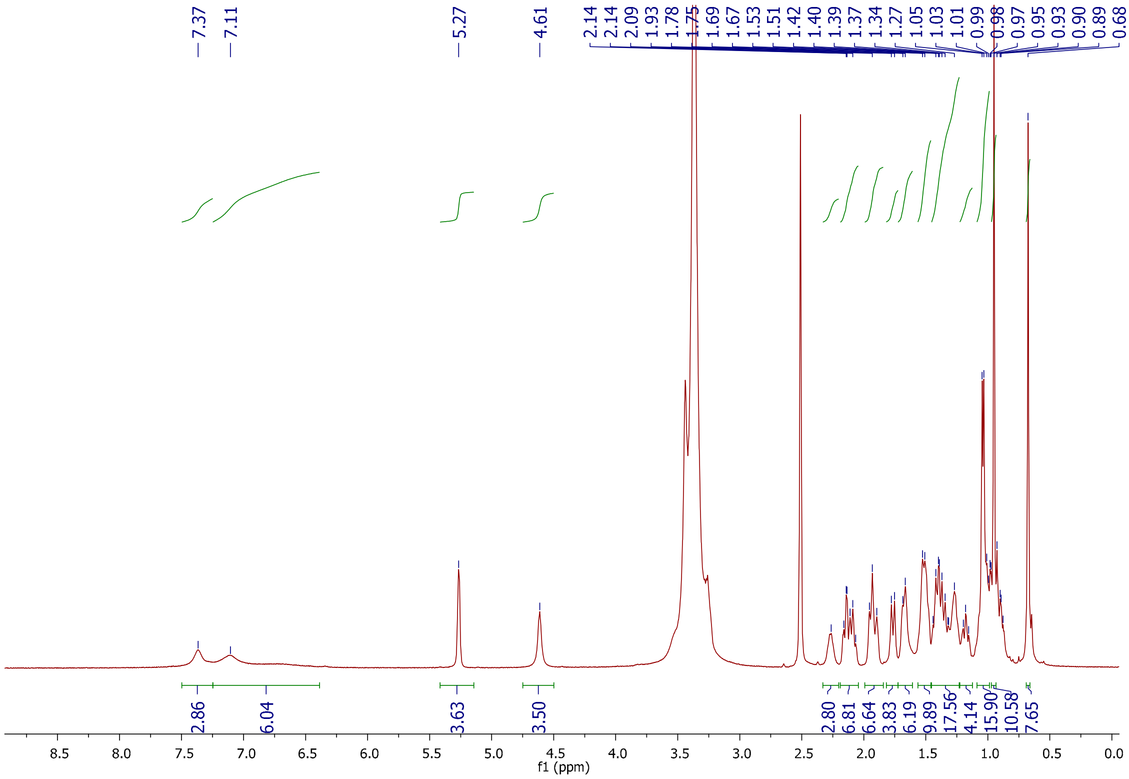

Figure S3. ^{1}H NMR spectrum of $ZnCl_2$(**H3**)$_2$ in DMSO-*d6*.

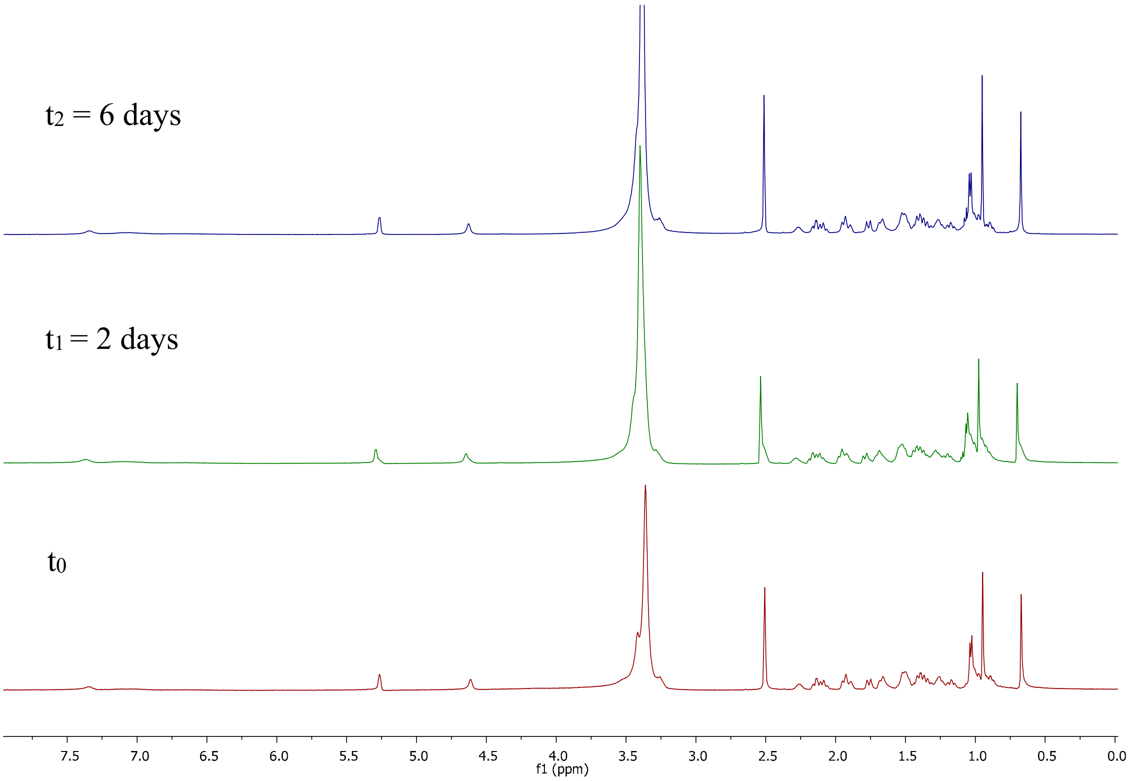

Figure S4. Stability study of $ZnCl_2$(**H3**)$_2$ by ^{1}H NMR in DMSO-*d6* as solvent.
